# Nexrutine^®^ preserves muscle mass similar to exercise in prostate cancer mouse model

**DOI:** 10.14814/phy2.14217

**Published:** 2019-08-27

**Authors:** Darpan I. Patel, Derek Wallace, Kira Abuchowski, Paul Rivas, Amber Gallegos, Nicolas Musi, Addanki Pratap. Kumar

**Affiliations:** ^1^ School of Nursing University of Texas Health Science Center at San Antonio San Antonio Texas; ^2^ Barshop Institute for Aging University of Texas Health Science Center at San Antonio San Antonio Texas; ^3^ Mays Cancer Center at UT Health San Antonio San Antonio Texas; ^4^ Department of Urology, School of Medicine University of Texas Health Science Center at San Antonio San Antonio Texas

**Keywords:** atrophy, inflammation, natural product, neoplasm of the prostate, proteolysis‐inducing factor

## Abstract

Muscle loss is a debilitating side effect to prostate cancer (PCa) experienced by nearly 60% of men. The purpose of this study was to test the hypothesis that Nexrutine^®^, a bark extract from the *Phellodendrum amurense*, can protect against prostate cancer induced muscle loss in a similar manner as exercise, using the transgenic adenocarcinoma of mouse prostate (TRAMP) model. Forty‐five, 8‐ to 10‐week old TRAMP mice were randomized to either control, Nexrutine^®^ (600 mg/kg pelleted in chow) or exercise (voluntary wheel running). Mice were serially sacrificed at weeks 4, 8, 12, and 20, at which time either the left or right gastrocnemius muscle was harvested, weighted, and frozen. Proteolysis inducing factor (PIF), ubiquitin, and NF‐κB concentrations were quantified using ELISA kits. Nexrutine^®^ and exercise were equally able to protect TRAMP mice against PCa‐induced muscle loss (*P* = 0.04). Both interventions decreased intramuscular PIF concentrations at 20 weeks compared to control (*P* < 0.05). A treatment effect was also observed when all time points were combined with exercise significantly lowering PIF concentrations (*P* < 0.01). Exercise significantly lowered intramuscular ubiquitin concentrations in weeks 4, 8, and 20 compared to control mice (*P* < 0.001). A treatment effect was also observed with exercise significantly lowering ubiquitin compared to control mice (*P* < 0.001). No significant changes were observed for NF‐κB. The results of this investigation demonstrate that PCa‐induced muscle loss can be attenuated with the herbal supplement Nexrutine^®^. This investigation provides preliminary evidence to support continued research into Nexrutine^®^ as a potential exercise analog in protecting against muscle loss.

## Introduction

In men with prostate cancer (PCa), a large percentage of men will suffer from muscle loss, a debilitating adverse effect, induced both by chronic illnesses or treatment paradigms (Cheung et al., 2018). The loss of skeletal muscle is vastly detrimental due to the integral roles skeletal muscle has in preserving a patient’s structure, locomotion, blood flow, respiration, and overall survivability (Pak et al., 2019). As a result, muscle loss leads to a reduction in muscle function, strength, endurance, and quality of life (Galvao et al., 2009).

Many proteins, either tumor‐derived or host‐derived, initiate the inflammatory response stimulating proteolysis and muscle loss (Tracey et al., 1988; Todorov et al., 1996; Li et al., 1998; Li and Reid, 2000; Schaap et al., 2006; Senf et al., 2013). Proteolysis‐inducing factor (PIF) is a tumor‐secreted protein that has been hypothesized to be a catalyst for stimulating protein degradation in muscle (Lorite et al., 1998; Cabal‐Manzano et al., 2001; Tisdale, 2002). Once bound to its receptor (PIFR), PIF activates IκB kinase (IKK) complex (consisting of IKKα, IKKβ, and NF‐κB essential modulator [NEMO]), promoting phosphorylation of IκB and subsequent activation of NF‐κB transcription factor subunits (Wyke and Tisdale, 2005). Downstream stimulation of the ubiquitin–proteasome system (UPS) leads to proteolysis and muscle loss (Li et al., 1998).

Pharmacological interventions have been studied to combat atrophic signaling but have been shown to be mostly ineffective (Gullett et al., 2011; Inui, 2012; Lobina et al., 2014; Ma et al., 2014; Aoyagi et al., 2015). Exercise is a viable treatment option for muscle loss and has shown to elicit muscle preservation while preventing proteolysis (Petersen and Pedersen, 2005). Unfortunately, the majority of cancer patients fail to follow the recommended daily guidelines of exercise (Bellizzi et al., 2005; Coups and Ostroff, 2005). Reduction in function due to cancer and its treatment, coupled with a hypoactive lifestyle creates an environment where physical exercise may not be a feasible treatment modality. We are prompted to seek an alternative treatment option for the population of individuals who lack an appropriate treatment option.

As many of the current treatment options include herbal medicines (i.e., natural products [Gullett et al., 2011]), we postulate the role that an investigational natural product, Nexrutine^®^, may have in preventing cancer‐associated muscle loss. Nexrutine^®^, a bark extract from the *Phellodendrum amurense*, is native to eastern Asia where it has been used in Chinese medicine to treat ailments including joint pain, psoriasis, and gastroenteritis (Cuellar et al., 2001; Hussain et al., 2015). Previous work by our group has demonstrated the anticancer effects of Nexrutine^®^ in various tumor models (Garcia et al., 2006; Kumar et al., 2007; Ghosh et al., 2010; Kumar et al., 2010; Gong et al., 2014; Hussain et al., 2015). This leads us to hypothesize that Nexrutine^®^ may be beneficial in protecting against muscle loss associated with tumors. Berberine and related compounds of Nexrutine^®^ have been shown to inhibit nuclear factor kappa‐light‐chain‐enhancer of activated B cells (NF‐kB) signaling and subsequent activation stemming from this pathway (Muralimanoharan et al., 2009). We hypothesize that the properties of Nexrutine^®^ can be transferrable to skeletal muscle maintenance by reducing proteolytic signaling. The objective of this study was to determine if Nexrutine^®^ can attenuate prostate cancer‐associated muscle loss in the same manner as exercise. In this manner, we aimed to evaluate Nexrutine^®^ as an exercise analog.

## Methods

### Study design

TRAMP Mice (*n* = 45) were equally randomized into three arms: control, exercise, and Nexrutine^®^. Three mice were sacrificed randomly from each of the groups in weeks 4, 8, and 12, and the remainder of the mice were sacrificed at week 20 (*n* = 6). At each sacrifice, a single gastrocnemius muscle was randomly selected and dissected, cleaned of debris, weighed, flash frozen, and stored for analysis. Additionally, prostate tumors were harvested, cleaned, and imbedded in 10% formalin for future histology analysis. All animal treatments and procedures were approved by the University of Texas Health Science Center at San Antonio’s Institutional Animal Care and Use Committee (IACUC).

### Animal model

Male transgenic adenocarcinoma of the mouse prostate (TRAMP) mice, between 8 and 10 weeks old, were purchased from Jackson Laboratories (Bar Harbor, ME). TRAMP mice are bred from a C57BL/6 background and spontaneously develop progressive forms of PCa. PCa within these mice intimately mirrors the pathology of that found in humans and make for a reliable model for this study (Anzo et al., 2008; Llaverias et al., 2010; Bonorden et al., 2012).

### Treatment groups

Mice randomized to the Nexrutine^®^ group were given 600 mg/kg of Nexrutine^®^ purchased from Interhealth (Benicia, CA) and pelleted into traditional chow by Dyetz, Inc. (Bethelem, PA). This concentration was used based on previous reported work regarding stability and concentration of Nexrutine^®^ in the diet pellets (Kumar et al., 2007). Mice randomized to the exercise group ran freely on a 15.5 cm diameter, low‐profile running wheel (Med Associates Inc., St. Albans, VT). Wheel revolutions were recorded continuously by an electronic sensor incorporated into the wheel and analyzed using the provided software. Control mice were maintained under normal conditions. All mice were housed individually to ensure accurate measurement of running wheel activity and food consumption. All mice followed a traditional light–dark cycle and were provided food and water *ad libitum*. Weekly measurements of food consumption and body weight were performed.

### Protein quantification

Gastrocnemius muscles were homogenized in phosphate‐buffered saline (PBS) solution relative to muscle mass. PIF and ubiquitin concentrations were quantified by using commercially available enzyme‐linked immunosorbent assay (ELISA) kits from MyBioSource Inc. (San Diego, CA). The sensitivities for the assays ranged from 0.1 ng/mL and 10 pg/mL, respectively. NF‐κB concentrations were quantified via a commercial ELISA kit purchased from Life Technologies (Frederick, MD) with a sensitivity of <17 pg/mL. Each sample was assayed in duplicate and averaged.

### Statistical analysis

Statistical analysis was completed using SPSS (IBM, Armonk, NY, USA) and presented graphically using GraphPad Prism (La Jolla, CA, USA). Comparison of the groups was done using a two‐way analysis of variance (ANOVA) with Tukey’s post hoc analysis. Pearson’s product moment correlation was performed to determine associations between variables. All data are expressed as mean ± standard deviation with significance set at *P* < 0.05.

## Results

### Intervention tolerance

Mice randomized to the exercise intervention were given continuous access to a running wheel. Wheel‐running data for the exercise group were measured in revolutions via electronic sensor attached to the running wheel. The mice within this group on average ran 13.6 ± 4.1 km/day. No significant differences were observed between mice in this group. Mice who were randomized to the Nexrutine^®^ intervention consumed 17.97 ± 1.24 g of Nexrutine^®^ imbedded chow per day. Analysis of relationships with Nexrutine^®^ consumption showed a negative correlation with regard to tumor mass (*r* = 0.74, *P* = 0.006). Comparison between the body and tumor masses between the three groups yielded no significant differences (Table 1).

**Table 1:** Animal characteristics.

	Control	Nexrutine^®^	Exercise
Body mass (g)	33.2 ± 7.28	31.5 ± 4.0	35.2 ± 8.5
Tumor mass (g)	4.9 ± 7.8	4.7 ± 6.0	7.2 ± 8.4
Weekly food consumption (g/week)	26.1 ± 3.6	18.1 ± 1.3*	28.2 ± 3.0
Running wheel activity (km/day)			13.6 ± 4.1

* Indicates significant differences between compared to Exercise group (*P* < 0.05).

### Nexrutine^®^ and exercise protect against skeletal muscle loss

**Figure 1:**
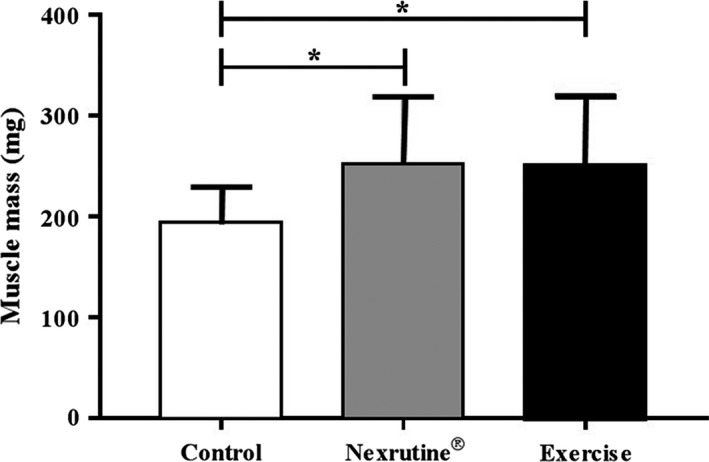
Nexrutine^®^ and exercise similarly preserve muscle mass in TRAMP mice. A treatment response was observed across the 20‐week intervention demonstrating that Nexrutine^®^ mirrors exercise in preventing muscle loss associated with a tumor burden. * indicates significance at *P* < 0.05.

At necropsy, a unilateral gastrocnemius was randomly selected for dissection and analysis. No significant differences were observed with relation to time points. Significant differences were observed (*F*(2,34) = 4.159, *P* = 0.024) with both Nexrutine^®^ (*P* = 0.04) and exercise (*P* = 0.04) mice having greater gastrocnemius mass compared to the control, respectively (Fig. 1). No correlations were found between muscle mass and distance ran or amount of Nexrutine^®^ consumed.

### Exercise downregulates PIF protein concentrations

**Figure 2:**
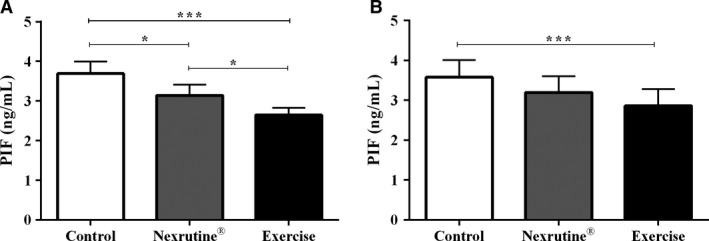
Exercise reduces intramuscular concentrations of proteolysis‐inducing factor. Time point analysis and treatment effect were analyzed. Analysis revealed that PIF was significantly lower in both Nexrutine^®^ and Exercise mice at the 20‐week time point only (A). All samples were pooled together and analyzed revealing a treatment effect for exercise (B). * indicates significance at *P* < 0.05; *** indicates significance at *P* < 0.001.

Intramuscular PIF concentrations were quantified following manufacturer’s recommended protocol in gastrocnemius homogenates at 4, 8, 12, and 20 weeks. Time point analysis revealed significance differences between groups at 8 (*F*(2, 7) = 5.36; *P* = 0.04) and 20 weeks (*F*(2, 10) = 22.85, *P* < 0.001). Post hoc analysis revealed no significant differences between the groups at 8 week. However, exercise was shown to decrease PIF concentrations in mice at 20 weeks (Fig. 2A) when compared with both the control (*P* < 0.001) and Nexrutine^®^ (*P* = 0.037) mice. Similar to exercise, Nexrutine^®^ elicited a decrease in PIF protein concentrations at 20 weeks (Fig 2A) compared to control (*P* = 0.033). A treatment effect was also observed when all time points were combined (*F*(2, 32) = 10.84, *P* < 0.001) with exercise significantly lowering PIF concentrations across the entire protocol (*P* < 0.001; Fig 2B). Pearson’s correlation analysis revealed consumption of Nexrutine^®^ to be inversely related to PIF concentrations (*r* = −0.77, *P* = 0.006). When all samples (*n* = 45) were combined to determine if there was a relationship between PIF and ubiquitin, a significant positive correlation was observed (*r* = 0.44, *P* = 0.008).

### Exercise reduces ubiquitin protein concentrations

**Figure 3:**
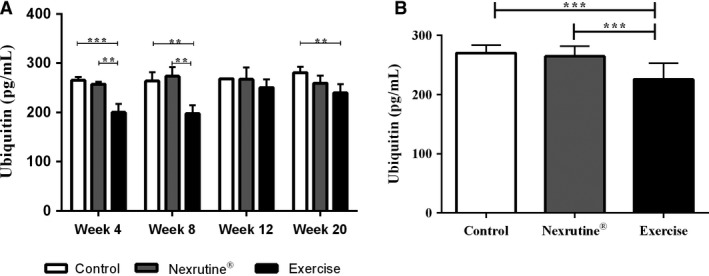
Exercise significantly decreases ubiquitin in tumor‐bearing mice. (A) Analysis of ubiquitin concentrations with regards to time point dissections between groups. Results show exercise was capable of reducing intramuscular ubiquitin concentrations at week 4, 8, and 20. (B) Overall treatment response for exercise was also observed when all samples were pooled together. ** indicates significance at *P* < 0.01; *** indicates significance at *P* < 0.001.

Intramuscular ubiquitin concentrations were quantified following manufacturer’s recommended protocol. Time point analysis of ubiquitin between groups is shown in Figure 3A. Time point analysis yielded significance differences in weeks 4 (*F*(2, 6) = 32.35, *P* < 0.001), 8 (*F*(2, 7) = 16.24, *P* = 0.002), and week 20 (*F*(2, 10) = 8.295, *P* = 0.008). Post hoc analysis revealed exercise significantly lowered ubiquitin concentrations compared to control and Nexrutine^®^ groups, respectively (*P* < 0.01). Overall treatment response was observed between the three groups (*F*(2, 38) = 19.55, *P* < 0.001; Fig. 3B). Post hoc analysis revealed exercise significantly reduced ubiquitin concentrations compared to both control and Nexrutine^®^, respectively (*P* < 0.0001).

### NF‐κB unresponsive to treatments

NF‐κB protein concentrations were quantified following manufacturer’s recommended protocol. Time point analysis and treatment response were conducted. No significant changes were observed with respect to time point and treatment response. A positive association was observed between NF‐κB and body mass (*r* = 0.34, *P* = 0.03) as well as NF‐κB and tumor mass (*r* = 0.33, *P* = 0.04).

## Discussion

Determining the effects of novel products on muscle mass and comparing these effects to exercise are clinically important in managing prostate cancer. Therefore, it is imperative that we identify products that can prevent the deleterious effects of tumor burden. Determining the positive outcomes in a treatment naïve group provides rational for continued study as prehabilitation prior to the initiation of androgen deprivation therapy, chemotherapy and/or radiation therapy can attenuate the underlying causes of cancer‐related fatigue and cancer‐induced cachexia often seen with more advanced cases. The primary findings of this study suggest that the natural product Nexrutine^®^ protects skeletal muscle against tumor‐associated decline in muscle mass. This is clinically important as these results show that intervening with Nexrutine^®^ can have similar effects as exercise in protecting skeletal muscle against tumor‐derived factors that lead to atrophy and muscle loss.

On the basis of our previous publications using Nexrutine^®^ (Muralimanoharan et al., 2009; Gong et al., 2014), we hypothesized the reduction of the NF‐kB signaling would yield benefits in the protection against muscle loss associated with advanced tumors. Our hypothesis was partially supported such that the Nexrutine^®^ group had greater muscle mass compared to controls with lower PIF concentrations at the end of the intervention. Our hypothesis that Nexrutine^®^ has the potential as an exercise analog is supported with both Nexrutine^®^ and exercise groups having similar outcomes in muscle mass. This suggests that both interventions independently elicited a preventative effect of muscle loss. This preservation of muscle mass is critical in the prevention of frailty and maintenance of physical function and quality of life in cancer patients. Contrary to our hypothesis, we did not observe any changes in intramuscular NF‐κB concentration.

A key protein of interest in this study was PIF, a tumor‐derived protein associated with initiating proteolysis in skeletal muscle (Lorite et al., 1998; Cabal‐Manzano et al., 2001; Lorite et al., 2001; Tisdale, 2002). The outcomes of our study demonstrated a positive correlation between intramuscular PIF and ubiquitin concentrations, supporting published reports on the significance of PIF in muscle loss (Khal et al., 2005). A previous study by de Lima et al reported anaerobic exercise decreased PIF expression in tumor tissue after exercise (Lima et al., 2008). Both Nexrutine^®^ and exercise, respectively, were capable of reducing intramuscular PIF concentration during the intervention. However, only exercise was capable of reducing ubiquitin concentrations. Therefore, further research is required to understand Nexrutine^®^’s role in attenuating proteolysis and muscle loss.

Unlike exercise, Nexrutine^®^ did not evoke an overall treatment effect. A late‐stage effect was observed in the Nexrutine^®^ fed group evident by significantly lower PIF concentrations at the 20‐week time point. This late significance at 20 weeks suggests that the intervention’s effect on PIF may be time‐dependent. An interesting observation was made in this study in that a negative association was seen with Nexrutine^®^ consumption and PIF concentrations, suggesting a potential dose effect. Future research should consider measuring the treatment of different doses of Nexrutine^®^ to identify an ideal therapeutic dose.

Attempts to block PIF‐associated muscle loss are at the heart of many therapies prescribed to combat cancer‐related cachexia (Tisdale, 2002). Much of the therapies prescribed have not shown a positive effect on muscle mass the way Nexrutine^®^ did in this study. Both fish oil (Wigmore et al., 1996) and eicosapentaenoic acid (Wigmore et al., 2000) have been reported to stabilize muscle mass in conditions of cachexia. When combined with a nutritional intervention, fish oil was shown to increase muscle mass in cachectic cancer patients (Barber et al., 1999). Similarly, a novel leucine metabolite, b‐hydroxy‐b‐methulbutyrate increases fat‐free mass in cachectic cancer patients as well (May et al., 2002). The mechanisms of action remain unknown, however, given the nature of cachexia to be a pro‐inflammatory condition, these therapies likely exert an anti‐inflammatory state.

Contrary to our hypothesis, neither exercise nor Nexrutine® were able to reduce transcription factor NF‐κB concentrations in the gastrocnemius. This points to the potential for alternative transcription factors that are targets in this model, like STAT3 (Heinrich et al., 1998; Fischer and Hilfiker‐Kleiner, 2007). STAT3 phosphorylation, nuclear localization, and target gene expression are activated in cancer cachexia. Suppression of STAT3 has the potential to protect muscle protein loss (Bonetto et al., 2012; Silva et al., 2015). Nexrutine^®^ has been demonstrated to downregulate STAT3 (Gong et al., 2014). Although not measured in this study, STAT3 may be the mediator by which Nexrutine^®^ exerts its protective effect warranting further investigation.

Independent of the late‐stage PIF reductions, exercise was able to significantly lower intramuscular ubiquitin concentrations across the entire protocol with the exception of the samples collected at week 12. Ubiquitin and the ubiquitin–proteasome pathway plays a central role in the muscle loss associated with a tumor burden. Many studies in cancer patients with weight loss indicative of cachexia have suggested that the ubiquitin–proteasome pathways in skeletal muscle is highly active (Williams et al., 1999). Overactivation of this pathway has been associated with oxidative stress leading to a breakdown of myofibrillar proteins (Powers et al., 2005; Tisdale, 2005; Moylan and Reid, 2007). Aerobic exercise has been demonstrated to protect against muscle loss due to its effect on oxidative stress, counteracting the metabolic impairments and muscle atrophy associated with tumor burden (Alves et al., 2015). The results of our study support these results and provide further evidence that aerobic exercise attenuates muscle loss by decreasing intramuscular concentrations of ubiquitin in a tumor model. This reduced ubiquitin concentration likely protected the gastrocnemius muscle from proteolysis and subsequent muscle atrophy by slowing down the active ubiquitin–proteasome system.

## Limitations

The positive outcomes of this study are not without limitations. The length and design of the protocol may inherently be a limitation to this study. Ideal length of training to induce an exercise phenotype is traditionally 6‐8 week (Kregel et al., 2006). Based on our experience, animal activity decreased between week 12 and 20, likely due to tumor mass size. This increased mass may impede physical performance and impacting late‐stage outcomes. An interesting future study would include a cross over between exercise and Nexrutine^®^ supplementation at such a time that tumor mass limits physical performance. Furthermore, including serial sacrifices reduces statistical power the various time points, limiting our interpretations of the outcomes. Although significant difference were observed as the different time point (i.e., 4, 8, 12, and 20 weeks), future research should include a sample size with statistical power at each time point to determine if these outcomes are repeatable. The benefits of performing serial sacrifices in this exploratory study provides valuable information on the timing of future protocols and provides an insight into the variability in proteins impacted by our interventions. The lack of a nontumor‐bearing control group limits our ability to determine if the tumor burden experienced by these transgenic mice reduces muscle mass. Others have indicated that the presence of tumor does reduce muscle though in different tumor models (Baumfalk et al., 2019). Furthermore, the protection against atrophy based on wet tissue weight of gastrocnemius muscles does not account for the degree of edema that may be present. Normalizing tissue mass to either bone length or assessing muscle cross sectional area may provide better insight into the effects of our interventions. Finally, we are limited in our understanding of the systemic effects of our interventions on PIF and NFκB because circulating concentrations were not measured. Future research should address these limitations to provide better insights on the effects of Nexrutine^®^ and exercise in tumor challenged models.

## Conclusions

This study provides evidence that exercise and Nexrutine^®^ have great potential to reduce skeletal muscle loss induced by cancer. These treatments can be very beneficial for clinical medicine not only due to the lack of standardized treatment for cachexia, but the ideology that both treatments are multifocal when compared with unifocal pharmacological agents.

## Conflict of interest

None declared.
